# Healthcare utilisation among migrants in the Netherlands’ unique hybrid healthcare system: the HELIUS study

**DOI:** 10.1136/bmjgh-2025-020024

**Published:** 2026-03-16

**Authors:** Mary Cassidy, Charles Agyemang, Henrike Galenkamp, Eric Moll van Charante, Felix P Chilunga

**Affiliations:** 1 Department of Public and Occupational Health, Amsterdam Public Health Research Institute, Amsterdam UMC-Locatie AMC, University of Amsterdam, Amsterdam, The Netherlands; 2 Department of General Practice, Amsterdam UMC, University of Amsterdam, Amsterdam, The Netherlands

**Keywords:** Global Health, Health Services Accessibility

## Abstract

**Background:**

Migrants in Europe often face barriers to healthcare, contributing to poorer health outcomes. While healthcare utilisation has been studied within Beveridge systems (eg, the UK) and Bismarck systems (eg, Germany), less is known about healthcare use by migrants within the Netherlands’ hybrid model. The Dutch model combines a Bismarckian base with Beveridge-like supplements. We investigated the use of general practitioner (GP) services, specialist care, allied health services and complementary medicine among major migrant groups in Amsterdam (South Asian Surinamese, African Surinamese, Ghanaian, Turkish and Moroccan origin) compared with the Dutch-origin population.

**Methods:**

We used pre-pandemic Healthy Life in an Urban Setting data (2015; n=21 614) to avoid biases from intra-COVID-19 and post-COVID-19 healthcare disruptions/alterations. Structured questionnaires assessed healthcare use and reasons for seeking care. Poisson regression with a log link and robust (sandwich) standard errors examined associations between migration background and healthcare utilisation, adjusting for demographics, acculturation, health literacy, lifestyle and chronic conditions. Sensitivity analyses explored motivations for care use and overall health status.

**Results:**

All migrant groups reported higher or similar use of GP services compared with the Dutch-origin population. Most migrant groups (except Ghanaians) also reported higher or similar use of specialist and allied health services. Conversely, use of complementary medicine was higher among Dutch-origin participants than among migrants.

**Conclusion:**

Many migrant groups in Amsterdam show higher use of mainstream healthcare services compared with previous reports from other European settings. Further studies should examine and dissect these patterns to inform improvements in other European settings.

**WHAT IS ALREADY KNOWN ON THIS TOPIC**

- Migrants in Europe often experience lower access to healthcare in Beveridge-type (eg, UK, Spain) and Bismarck-type systems (eg, Germany, France).
- Evidence on healthcare utilisation within hybrid systems, such as the Dutch healthcare system, is limited.

**WHAT THIS STUDY ADDS**

- In Amsterdam, many migrant groups reported similar or higher general practitioner use compared with Dutch-origin individuals, independent of demographic, cultural and health-related factors.
- Specialist and allied health service use was comparable or higher in many migrant groups, except among Ghanaian-origin participants.

**HOW THIS STUDY MIGHT AFFECT RESEARCH, PRACTICE OR POLICY**

- Many migrant groups in Amsterdam show higher use of mainstream healthcare services compared with previous reports from other European settings.
- Further studies could examine and dissect these patterns to inform improvements in other European settings.

## Introduction

 International migration from low- and middle-income countries (LMICs) to high-income regions like Europe has risen substantially in recent decades.^1^ Addressing migrant health has become a critical public health issue,^2^ as migrants from LMICs often face a disproportionately high disease burden compared with native-born populations in their host countries.^3^ This disparity spans a wide range of health conditions, including cardiometabolic diseases (eg, coronary artery disease, type 2 diabetes and obesity),^3^,^5^ mental health challenges (eg, depression)^6^ and infectious diseases (eg, tuberculosis).^7^

These elevated health risks are compounded by significant barriers to healthcare access, creating a complex landscape of inequity.^8 9^ Migrants face many obstacles in accessing healthcare, including language barriers that can hinder their understanding of the healthcare system,^8 9^ as well as cultural differences in health care-seeking behaviour.^9 10^ Legal and financial constraints,^9 11 12^ often linked to uncertain healthcare entitlements or limited awareness of subsidies, add to these challenges.^9^ Navigating complex healthcare systems in an unfamiliar language and bureaucratic setting can be overwhelming, especially when discrimination erodes trust and discourages healthcare use.^9 13^

To date, migrant healthcare access has primarily been studied in two prevalent healthcare models: the Beveridge and Bismarck models.^14 15^ Within the Beveridge model,^15^ as seen in the UK and Sweden,^16^ tax-funded universal coverage minimises out-of-pocket expenses.^14^ Within the Bismarck model,^15^ found in countries like Germany and France,^17^ social insurance schemes link healthcare coverage to employment.^14^ Within these healthcare models,^14 15^ migrants often face healthcare access rates 2–3 times lower than those of the majority population, largely due to systemic challenges related to navigation, awareness and cultural barriers.^18^,^22^

The Netherlands presents a unique case with its dual healthcare system introduced in 2006.^23^ The Dutch system follows a “managed competition” model—a hybrid that combines elements of both the Bismarck and Beveridge models.^23^ All residents are mandated to have basic health insurance, which is offered by private insurers competing on price and supplemental coverage, promoting market efficiency within a universal framework.^23^ Unlike the employer-tied insurance of the Bismarck model or the fully tax-funded approach of the Beveridge model, the Dutch system integrates individual mandates with regulated private insurance.^23^ This ensures universal access while encouraging cost control and consumer choice.^23^ The government plays a key role in setting healthcare priorities, regulating insurers and ensuring quality, creating a balanced system that leverages both public and private mechanisms.^23^

Even though the system is aimed at universal access, navigating the Dutch healthcare system may be challenging for migrants unfamiliar with private insurance options, deductibles and co-payments, especially those with limited financial literacy or language skills.^23^ The reliance on private insurance could introduce inequities, as migrants with precarious legal status or limited financial resources may struggle to afford premiums or access certain services.^23^ The fragmented structure of the Dutch model, which requires migrants to interact with multiple entities—private insurers, general practitioners (GPs) who act as gatekeepers for specialist referrals, and government agencies for subsidies—may add complexity to healthcare access.^23^ Migrants may face administrative burdens related to understanding insurance options, applying for subsidies and managing deductibles, which can delay or deter timely care.^23^

While these structural hurdles exist, the system offers notable advantages. All residents, regardless of employment or immigration status, are eligible for basic health insurance, with government subsidies available to support low-income individuals.^23^ This provision is essential for migrants who may lack stable employment or access to employer-based insurance in other contexts. The basic package covers a wide array of services, including GP visits, specialist consultations, hospital care, mental health and maternity care.^23^

Despite these distinct structural features, evidence on how migrants use different types of healthcare services within the Dutch system remains limited. Previous studies in the Netherlands were small and focused on primary care in two migrant groups only (Turks and Moroccans),^24^ leaving gaps in understanding how broader groups of migrants use the wider healthcare landscape, including specialist, allied health and alternative care.

We therefore assessed the utilisation of various types of care—including GPs, specialist care, allied health services and complementary medicine—among diverse migrant populations of South-Asian Surinamese, African Surinamese, Ghanaian, Turkish and Moroccan origin, compared with the Dutch-origin population within the Dutch healthcare system (particularly in Amsterdam). We also assessed the reasons for the utilisation of these services and the role of health status.

## Methodology

### Study design and population

This study is part of the Healthy Life in an Urban Setting (HELIUS) study, a multi-ethnic prospective cohort based in Amsterdam, the Netherlands, focusing on cardiovascular diseases, mental health outcomes, infectious diseases and access to healthcare.^25^ Data were collected at two time points: baseline (2015) and follow-up (2021).^25 26^ For this analysis, only baseline data were used, as the follow-up period coincided with the COVID-19 pandemic—a time of significant disruption to the normal functioning of the healthcare system. Routine healthcare access was altered, services were delayed or reprioritised, and healthcare-seeking behaviour changed considerably. As such, healthcare utilisation data were not collected during the follow-up period, as it would not have been representative of a normally functioning healthcare system.

A detailed description of the HELIUS study has been published elsewhere.^25 26^ In brief, the HELIUS study recruited 24 789 participants at baseline, comprising individuals of Dutch origin as well as those from the six largest migrant groups in Amsterdam: African Surinamese, South-Asian Surinamese, Turkish, Moroccan and Ghanaian origin populations, aged 18 to 70 years. Participants were randomly selected from the Amsterdam municipal register, with stratification by ethnic group. A total of 49 952 individuals were contacted. Among those contacted, 24 789 agreed to participate (50%).^26^ Participation among contacted individuals varied by migration background: 60% among Dutch-origin individuals, 41% among Surinamese-origin individuals, 41% among Turkish-origin individuals, 43% among Moroccan-origin individuals and 61% among Ghanaian-origin individuals.^26^ All participants completed a self-administered questionnaire and underwent a physical examination, during which biological samples were collected. All measurements were conducted according to standardised protocols.^25 26^

### Migration background

Migration background was classified according to Statistics Netherlands, based on participants’ and their parents’ country of birth.^27^ Individuals were categorised as Dutch origin if they were born in the Netherlands with both parents also born in the Netherlands. Those with a migration background included individuals born abroad with at least one parent born abroad (first generation) or those born in the Netherlands with both parents born abroad (second generation). Stratified sampling resulted in six ethnic groups: Dutch origin, Surinamese origin (further classified after data collection as South-Asian or African Surinamese based on self-report), Turkish origin, Moroccan origin and Ghanaian origin.^25^

### Healthcare utilisation

Healthcare utilisation data were gathered through a structured questionnaire (online supplemental appendix 1). Participants were asked if they had consulted any of the following healthcare providers within the past 12 months: a GP, medical specialist (eg, cardiologist, dermatologist), allied health professional (eg, physiotherapist, dietician) or complementary medicine practitioner (eg, acupuncturist, homeopath, traditional healers).

Reasons for visiting the GP were not captured by the questionnaire. However, we captured self-rated health status. Self-rated health status was assessed using the first item of the SF-12 (12-Item Short Form Survey), a widely used general health questionnaire.^28^ Participants were asked: ‘In general, would you say your health is: excellent, very good, good, fair or poor?’ Responses were categorised into three groups: excellent/very good, good and fair/poor.

For consultations with a medical specialist, allied health professional or complementary medicine practitioner, participants were also asked to provide details about the specific type of provider they visited (eg, cardiologist, dermatologist, physiotherapist, etc online supplemental appendix 1).

### Other measurements

We also collected data on demographic characteristics (sex, age, occupational and educational level and marital status), lifestyle factors (smoking, alcohol consumption, physical activity), health literacy (Set of Brief Screening Question (SBSQ) scores), acculturation indicators (Dutch language proficiency, and Berry’s acculturation strategy) and chronic health conditions (diabetes, hypertension and chronic kidney disease (CKD)). A full description of these additional measures is provided in online supplemental appendix 2.

### Patient and public involvement

Community leaders were actively involved in the study’s design, recruitment and dissemination. Identified through local organisations (eg, churches, mosques) and supported by key figures, they co-developed recruitment strategies aligned with community needs. Engagement was further enhanced via local media (radio, TV) and collaborations with healthcare organisations. Researchers conducted on-site visits and mini clinics over 1–2 weeks to encourage participation. Study findings were shared through community briefs, local meetings and the study newsletter.

### Data analysis

Statistical analyses were performed in R. Baseline characteristics were presented as proportions for categorical variables and as means with SD for continuous variables. Missing data were minimal for all variables (<5%), except for occupation (16%, online supplemental appendix 3). Missing values were handled using multiple imputations by chained equations (MICEs). Ten imputed datasets were generated, and estimates were pooled using Rubin’s rules. The primary and secondary analyses were conducted on the imputed dataset (ie, the full analytical cohort), while complete-case analyses were performed as sensitivity analyses.

Age and sex standardised proportions for the four healthcare utilisation indicators, GP, specialist, allied health and complementary medicine use, were calculated for each migration background group using direct standardisation, with the total sample serving as the reference population.

Primary analyses examined associations between migration background and healthcare utilisation using Poisson regression models with a log link function and robust sandwich standard errors (*glm function, sandwich package*) to estimate prevalence ratios (PRs). Because the outcomes were common, PRs provide a less inflated measure of association than ORs. Models were adjusted for sociodemographic characteristics, lifestyle factors, acculturation indicators, health literacy and chronic health conditions. Model diagnostics included assessment of multicollinearity using variance inflation factors (<5), and model convergence was confirmed by successful fitting without estimation warnings. All presented models met these criteria.

Secondary exploratory analyses were conducted to evaluate the robustness and interpretation of the primary associations. For GP utilisation, stratified analyses by self-rated health status (fair or poor, good, very good or excellent) and by diabetes and hypertension status were performed to assess whether observed differences reflect underlying health needs. Differences in the specific types of specialists, allied health and complementary medicine services were assessed using χ^2^ tests to characterise patterns of service use and provide insight into potential reasons for utilisation across migration background groups. No formal adjustment for multiple testing was applied, as secondary analyses were considered exploratory.

In sensitivity analyses, primary models were conducted on the unimputed dataset and compared with the imputed dataset to rule out bias from multiple imputations. For all analyses, PRs with 95% CIs were reported. All statistical tests were two-tailed, with an alpha level of 0.05.

## Results

### Baseline characteristics

**Figure 1:**
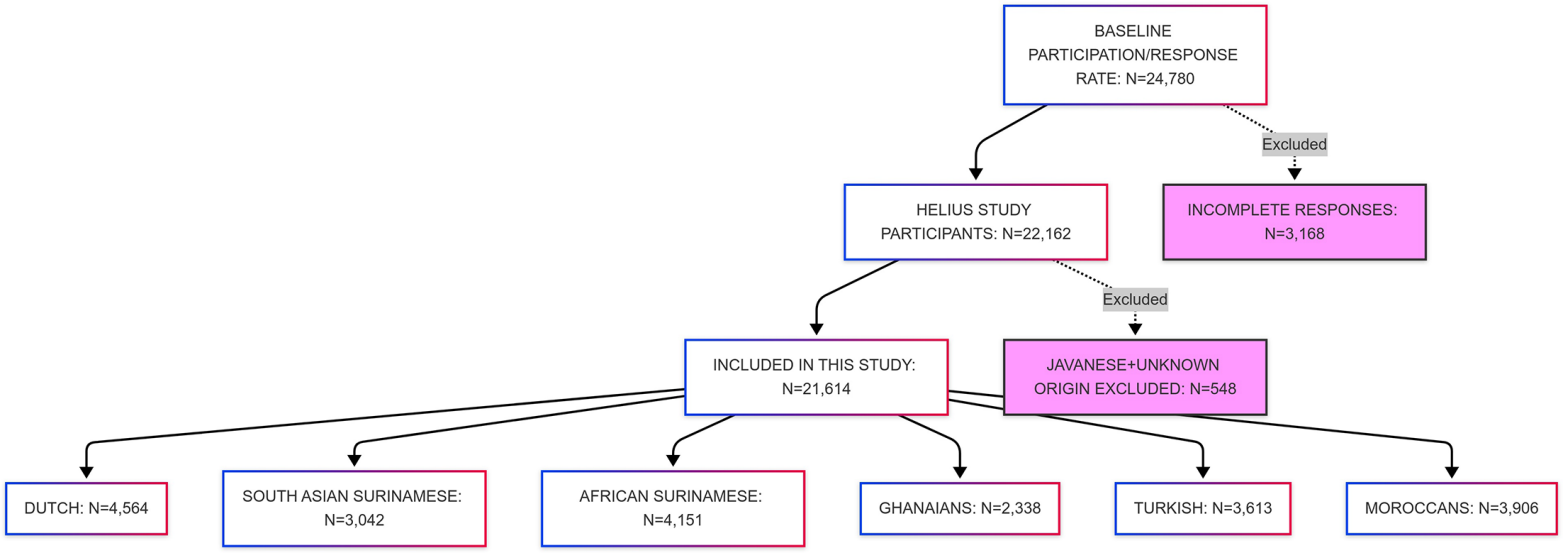
Flow chart of participation. HELIUS, Healthy Life in an Urban Setting.

Of the initial 24 780 participants, 22 162 filled in the questionnaire and completed a physical examination. Of these, 548 participants were excluded due to Javanese or unknown origin, resulting in a final sample of 21 614 participants. This final sample included Dutch (n=4564), South-Asian Surinamese (n=3042), African Surinamese (n=4151), Ghanaian (n=2338), Turkish (n=3613) and Moroccan (n=3906) origins (figure 1).

The mean age was 44 years, with 58% female. Marital/cohabiting rates were highest among Turkish (65%) and Moroccan (63%) groups, while 54% of African Surinamese participants were unmarried nor cohabiting together (table 1). Higher education was most common among Dutch participants (61%), whereas only 6% of Ghanaians and 15% of Turkish participants had higher education. Additionally, 60% of Dutch participants had higher/scientific occupations while only 4% of Ghanaians had higher/scientific occupations.

**Table 1:** Baseline characteristics of participants

Variables	Total n=21 614	Dutch origin n=4564	South-Asian Surinamese origin n=3042	African Surinamese origin n=4151	Ghanaian origin n=2338	Turkish origin n=3613	Moroccan origin n=3906
Age (years), mean (SD)							
	44.3 (13.2)	46.2 (14.1)	45.5 (13.4)	47.9 (12.5)	44.7 (11.2)	40.4 (12.2)	40.5 (12.9)
Sex, n (%)							
Male	9128 (42.2)	2089 (45.8)	1371 (45.1)	1616 (38.9)	905 (38.7)	1633 (45.2)	1514 (38.8)
Female	12 486 (57.8)	2475 (54.2)	1671 (54.9)	2535 (61.1)	1433 (61.3)	1980 (54.8)	2392 (61.2)
Marital status, n (%)							
Married partnership	8486 (39.3)	1726 (37.8)	1049 (34.5)	768 (18.5)	426 (18.2)	2216 (61.3)	2301 (58.9)
Cohabiting together	2345 (10.8)	915 (20.0)	311 (10.2)	445 (10.7)	432 (18.5)	132 (3.7)	110 (2.8)
Unmarried	7303 (33.8)	1476 (32.3)	1004 (33.0)	2252 (54.3)	796 (34.0)	765 (21.2)	1010 (25.9)
Divorced or separated	3049 (14.1)	360 (7.9)	584 (19.2)	620 (14.9)	660 (28.2)	409 (11.3)	416 (10.7)
Widow/widower	431 (2.0)	87 (1.9)	94 (3.1)	66 (1.6)	24 (1.0)	91 (2.5)	69 (1.8)
Occupational, n (%)							
Elementary	4104 (19.0)	94 (2.1)	358 (11.8)	301 (7.3)	1473 (63.0)	908 (25.1)	970 (24.8)
Lower	6671 (30.9)	752 (16.5)	1100 (36.2)	1484 (35.8)	553 (23.7)	1454 (40.2)	1328 (34.0)
Medium	5458 (25.3)	1084 (23.8)	909 (29.9)	1446 (34.8)	220 (9.4)	789 (21.8)	1010 (25.9)
Higher vocational/university	3936 (18.2)	1706 (37.4)	521 (17.1)	797 (19.2)	71 (3.0)	340 (9.4)	501 (12.8)
Scientific	1445 (6.7)	928 (20.3)	154 (5.1)	123 (3.0)	21 (0.9)	122 (3.4)	97 (2.5)
Educational level, n (%)							
No schooling	3861 (17.9)	152 (3.3)	440 (14.5)	233 (5.6)	670 (28.7)	1149 (31.8)	1217 (31.2)
Lower secondary	5693 (26.3)	655 (14.4)	1015 (33.4)	1490 (35.9)	934 (39.9)	896 (24.8)	703 (18.0)
Upper secondary	6291 (29.1)	1000 (21.9)	889 (29.2)	1477 (35.6)	589 (25.2)	1031 (28.5)	1305 (33.4)
Higher education vocational/university	5769 (26.7)	2757 (60.4)	698 (22.9)	951 (22.9)	145 (6.2)	537 (14.9)	681 (17.4)
Smoking, n (%)							
Yes	5194 (24.0)	1132 (24.8)	864 (28.4)	1313 (31.6)	106 (4.5)	1251 (34.6)	528 (13.5)
Never	12 112 (56.0)	1694 (37.1)	1762 (57.9)	2024 (48.8)	2038 (87.2)	1710 (47.3)	2884 (73.8)
Former	4308 (19.9)	1738 (38.1)	416 (13.7)	814 (19.6)	194 (8.3)	652 (18.0)	494 (12.6)
Alcohol consumption, n (%)							
Any consumption	10 932 (50.6)	4153 (91.0)	1713 (56.3)	2847 (68.6)	1115 (47.7)	816 (22.6)	288 (7.4)
No consumption	10 682 (49.4)	411 (9.0)	1329 (43.7)	1304 (31.4)	1223 (52.3)	2797 (77.4)	3618 (92.6)
Physical activity levels, n (%)							
Active	12 191 (56.4)	3448 (75.5)	1626 (53.5)	2535 (61.1)	1246 (53.3)	1508 (41.7)	1828 (46.8)
Not active	9423 (43.6)	1116 (24.5)	1416 (46.5)	1616 (38.9)	1092 (46.7)	2105 (58.3)	2078 (53.2)
Health literacy, n%							
Adequate subjective	18 333 (85.2)	4530 (99.3)	2812 (92.8)	4027 (97.2)	1499 (64.8)	2557 (71.3)	2908 (74.9)
Low subjective	3181 (14.8)	32 (0.7)	217 (7.2)	114 (2.8)	814 (35.2)	1031 (28.7)	973 (25.1)
Kidney function (eGFR <60 mL/min/1.73 m²), n (%)							
Yes	274 (1.3)	37 (0.8)	49 (1.6)	95 (2.3)	60 (2.6)	15 (0.4)	18 (0.5)
No	21 340 (98.7)	4527 (99.2)	2993 (98.4)	4056 (97.7)	2278 (97.4)	3598 (99.6)	3888 (99.5)
Diabetes, n (%)							
Yes	2343 (10.8)	165 (3.6)	592 (19.5)	498 (12.0)	273 (11.7)	370 (10.2)	445 (11.4)
No	19 271 (89.2)	4399 (96.4)	2450 (80.5)	3653 (88.0)	2065 (88.3)	3243 (89.8)	3461 (88.6)
Hypertension, n (%)							
Yes	8069 (37.3)	1354 (29.7)	1293 (42.5)	2098 (50.5)	1311 (56.1)	1060 (29.3)	953 (24.4)
No	13 545 (62.7)	3210 (70.3)	1749 (57.5)	2053 (49.5)	1027 (43.9)	2553 (70.7)	2953 (75.6)
Difficulty with Dutch language, n (%)							
Yes	7130 (41.8)	NA	715 (23.5)	527 (12.7)	1943 (83.1)	2159 (59.8)	1786 (45.7)
No	9920 (58.2)	NA	2327 (76.5)	3624 (87.3)	395 (16.9)	1454 (40.2)	2120 (54.3)
Berry’s acculturation							
Integration	13 110 (76.9)	NA	2437 (80.1)	3485 (84.0)	1724 (73.7)	2450 (67.8)	3014 (77.2)
Assimilation	674 (4.0)	NA	189 (6.2)	151 (3.6)	25 (1.1)	129 (3.6)	180 (4.6)
Separation	2982 (17.5)	NA	347 (11.4)	452 (10.9)	563 (24.1)	979 (27.1)	641 (16.4)
Marginalisation	284 (1.7)	NA	69 (2.3)	63 (1.5)	26 (1.1)	55 (1.5)	71 (1.8)
Self-rated health status							
Excellent/very good	5011 (23.2)	1927 (42.2)	474 (15.6)	874 (21.1)	684 (29.3)	501 (13.9)	551 (14.1)
Good	11 111 (51.4)	2207 (48.4)	1623 (53.4)	2339 (56.3)	1182 (50.6)	1878 (52.0)	1882 (48.2)
Fair/poor	5492 (25.4)	430 (9.4)	945 (31.1)	938 (22.6)	472 (20.2)	1234 (34.2)	1473 (37.7)
GP use: n (%)							
Yes	17 450 (80.7)	3493 (76.5)	2568 (84.4)	3509 (84.5)	1874 (80.2)	2876 (79.6)	3130 (80.1)
No	4164 (19.3)	1071 (23.5)	474 (15.6)	642 (15.5)	464 (19.8)	737 (20.4)	776 (19.9)
Specialist use, n (%)							
Yes	11 576 (53.6)	2325 (50.9)	1793 (58.9)	2428 (58.5)	1028 (44.0)	2027 (56.1)	1975 (50.6)
No	10 038 (46.4)	2239 (49.1)	1249 (41.1)	1723 (41.5)	1310 (56.0)	1586 (43.9)	1931 (49.4)
Allied health use, n (%)							
Yes	3802 (17.6)	715 (15.7)	576 (18.9)	757 (18.2)	382 (16.3)	667 (18.5)	705 (18.0)
No	17 812 (82.4)	3849 (84.3)	2466 (81.1)	3394 (81.8)	1956 (83.7)	2946 (81.5)	3201 (82.0)
Alternative health use, n (%)							
Yes	2836 (13.1)	846 (18.5)	427 (14.0)	501 (12.1)	238 (10.2)	431 (11.9)	393 (10.1)
No	18 778 (86.9)	3718 (81.5)	2615 (86.0)	3650 (87.9)	2100 (89.8)	3182 (88.1)	3513 (89.9)

Missing data were handled using multiple imputation (MICE); analyses were conducted on the fully imputed dataset (n=21 614).

eGFR, estimate the glomerular filtration rate; GP, general practitioner; NA, Not applicable in the Dutch group (eg, acculturation).

Smoking rates were highest among Turkish participants (35%) and lowest among Ghanaians (5%). Alcohol use was common among Dutch (91%) and low among Moroccans (7%), while being physically active was highest among the Dutch (76%) and lowest among the Turks (42%). Health literacy was highest among the Dutch (99%) and lowest among Turks (71%). Decreased kidney function was highest among Ghanaians (2.6%) and lowest in the Turkish and Moroccans (0.5%). Diabetes was most prevalent in South-Asian Surinamese (20%) and lowest in Dutch (4%), while hypertension was highest in Ghanaian (56%) and African Surinamese (51%) groups (table 1).

**Physical activity** was categorised as “Active” or “Not Active” according to the **SQUASH (Short Questionnaire to Assess Health-Enhancing Physical Activity**) which assesses physical activity based on frequency, duration and intensity of activities in daily life.

**Health literacy** was assessed using the SBSQ. The three-item SBSQ evaluates reading assistance, confidence in completing medical forms and difficulty understanding medical information. Responses were scored on a Likert scale and summed. A score of ≤2 indicates inadequate health literacy, while ≥3 suggests adequate health literacy.

**Kidney function** was assessed using the race-free CKD-EPI equation (2021) to estimate the glomerular filtration rate (eGFR) based on serum creatinine, age and sex. An eGFR of <60 mL/min/1.73 m² was classified as decreased kidney function while an eGFR of 60 mL/min/1.73 m² or higher was classified as normal kidney function.

**Diabetes** was defined according to the **WHO criteria** including fasting plasma glucose ≥126 mg/dL (7.0 mmol/L) or use of diabetes medication or previous doctor diagnosis.

**Hypertension** was defined by **WHO standards** including systolic blood pressure ≥140 mm Hg diastolic blood pressure ≥90 mm Hg or use of antihypertensive medication or previous doctor diagnosis.

**Difficulty with the Dutch language** was measured based on self-reported ease of understanding, speaking, reading and writing in Dutch, classified as “Yes” (difficulty) or “No” (no difficulty).

**Acculturation** is measured according to **Berry’s Acculturation Model** which includes Integration (maintaining one’s original culture while actively participating in the larger society), Assimilation (adopting the dominant culture and discarding one’s original culture), Separation (retaining one’s original culture while avoiding engagement with the larger society) and Marginalisation (disconnected from both one’s original culture and the dominant culture).

**Self-rated health status** was assessed using the first item of the SF-12, a widely used general health questionnaire. Participants were asked: ‘In general, would you say your health is: excellent, very good, good, fair or poor?’ Responses were categorised into three groups: excellent/very good, good and fair/poor.

**Healthcare utilisation** includes self-reported use of specific services within the past 12 months such as General Practitioner (**GP**) use, **Specialist use** (eg, cardiology, dermatology), **Allied Health use** (eg, physical therapy, dietetics, occupational therapy) and **Alternative Health use** (eg, acupuncture, homeopathy, naturopathy).

### Age and sex-adjusted proportion of healthcare use

**Figure 2:**
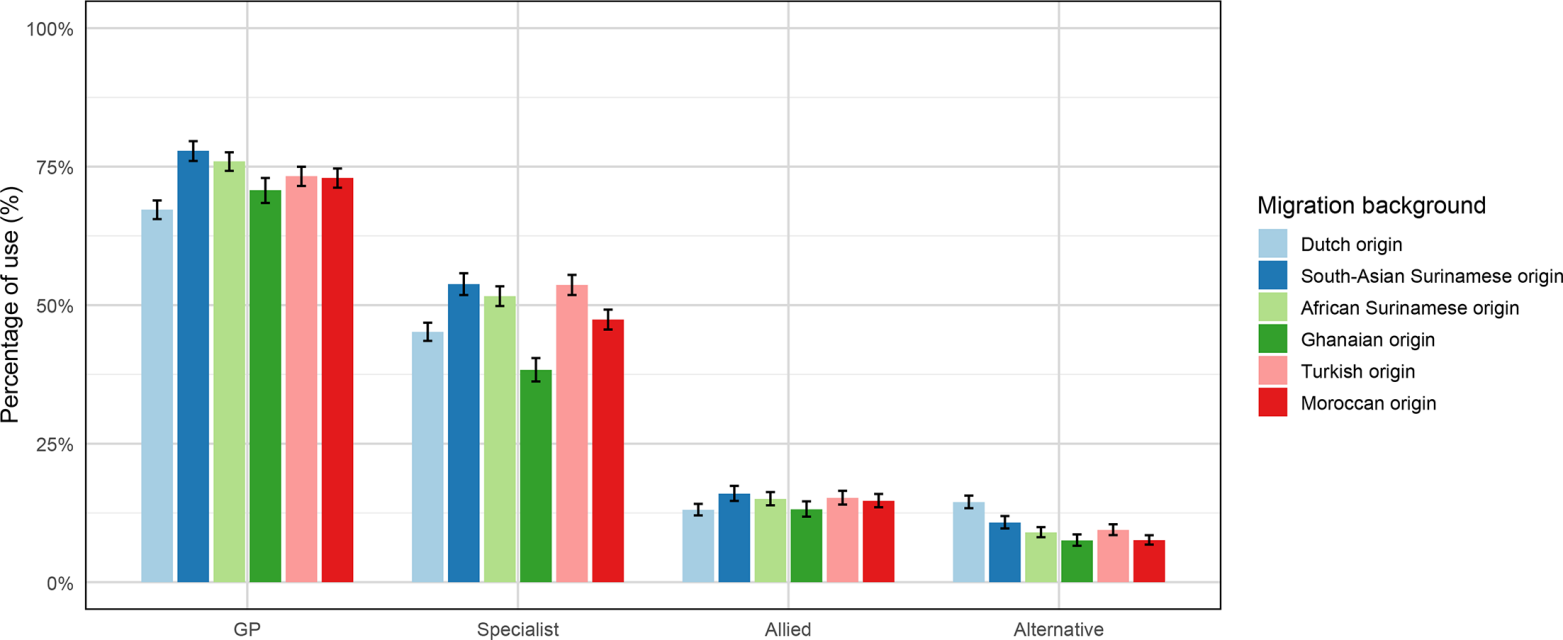
Age and sex-adjusted proportions of healthcare use among migrants and non-migrants. GP, general practitioner.

Age and sex-adjusted proportions (figure 2) revealed that healthcare utilisation among migrant groups in Amsterdam (the Netherlands) was generally higher than or equal to that of the Dutch population. GP use was generally high, ranging from 65% to 78%, with all migrant groups reporting higher utilisation than the Dutch population. South Asian Surinamese had the highest proportion. Specialist use varied between 38% and 53%, with migrant groups again surpassing the Dutch (except Ghanaians). African Surinamese, Moroccans and Turkish individuals showed the highest utilisation. Allied health services were used by 12% to 15% of individuals, with South Asian Surinamese individuals reporting the highest use. Alternative health services saw lower overall use in migrant populations, ranging from 7% to 14%, with the Dutch reporting highest use (figure 2).

### Migration background and GP service use

We found that South Asian Surinamese individuals (adjusted PR (aPR): 1.09, 95% CI 1.06 to 1.12) and African Surinamese individuals (aPR: 1.07, 95% CI 1.05 to 1.13), Turkish (aPR: 1.05, 95% CI 1.02 to 1.11) and Moroccan (aPR: 1.06, 95% CI 1.03 to 1.09) backgrounds are more likely to use GP services compared with those of Dutch origin. On the other hand, we found that individuals with Ghanaian origin (aPR: 1.02, 95% CI 0.99 to 1.15) exhibit GP utilisation patterns like those of Dutch origin after considering demographics, lifestyle factors, health literacy and chronic non-communicable diseases (table 2, online supplemental appendix 4).

**Table 2:** Associations between migration background and use of health services

	Model 1	Model 2	Model 3	Model 4	Model 5
	PR (95% CI)	PR (95% CI)	PR (95% CI)	PR (95% CI)	PR (95% CI)
General practitioner use					
Dutch origin	Reference	Reference	Reference	Reference	Reference
South Asian Surinamese origin	1.10 (1.08 to 1.13)	1.10 (1.08 to 1.13)	1.09 (1.07 to 1.12)	1.11 (1.08 to 1.13)	**1.09 (1.06 to 1.12)**
African Surinamese origin	1.10 (1.08 to 1.13)	1.08 (1.06 to 1.11)	1.08 (1.05 to 1.10)	1.09 (1.06 to 1.11)	**1.07 (1.05 to 1.10)**
Ghanaian origin	1.05 (1.02 to 1.07)	1.04 (1.01 to 1.07)	1.01 (0.98 to 1.04)	1.03 (1.00 to 1.06)	1.02 (0.99 to 1.05)
Turkish origin	1.04 (1.01 to 1.06)	1.06 (1.04 to 1.09)	1.04 (1.02 to 1.07)	1.05 (1.02 to 1.08)	**1.05 (1.02 to 1.08)**
Moroccan origin	1.05 (1.03 to 1.07)	1.06 (1.04 to 1.08)	1.04 (1.02 to 1.07)	1.06 (1.03 to 1.09)	**1.06 (1.03 to 1.09)**
Medical specialist use					
Dutch origin	Reference	Reference	Reference	Reference	Reference
South Asian Surinamese origin	1.16 (1.11 to 1.21)	1.16 (1.12 to 1.21)	1.13 (1.08 to 1.18)	1.15 (1.10 to 1.21)	**1.11 (1.06 to 1.16)**
African Surinamese origin	1.15 (1.11 to 1.20)	1.12 (1.08 to 1.17)	1.09 (1.04 to 1.13)	1.10 (1.06 to 1.15)	**1.07 (1.03 to 1.12)**
Ghanaian origin	0.86 (0.82 to 0.91)	0.87 (0.82 to 0.92)	0.85 (0.80 to 0.91)	0.88 (0.82 to 0.94)	**0.85 (0.80 to 0.91)**
Turkish origin	1.10 (1.06 to 1.15)	1.17 (1.12 to 1.22)	1.16 (1.11 to 1.22)	1.15 (1.09 to 1.21)	**1.14 (1.08 to 1.20)**
Moroccan origin	1.00 (0.95 to 1.04)	1.04 (1.00 to 1.09)	1.03 (0.98 to 1.09)	1.04 (0.98 to 1.10)	1.03 (0.98 to 1.09)
Allied health services use					
Dutch origin	Reference	Reference	Reference	Reference	Reference
South Asian Surinamese origin	1.22 (1.10 to 1.35)	1.21 (1.10 to 1.34)	1.04 (0.94 to 1.16)	1.11 (1.00 to 1.24)	1.09 (0.98 to 1.21)
African Surinamese origin	1.17 (1.06 to 1.28)	1.14 (1.04 to 1.26)	1.00 (0.90 to 1.10)	1.04 (0.94 to 1.15)	1.02 (0.92 to 1.13)
Ghanaian origin	1.03 (0.92 to 1.16)	1.01 (0.90 to 1.13)	0.72 (0.63 to 0.83)	0.86 (0.75 to 0.99)	**0.84 (0.73 to 0.97)**
Turkish origin	1.16 (1.06 to 1.29)	1.16 (1.05 to 1.28)	1.01 (0.90 to 1.13)	1.00 (0.89 to 1.14)	1.00 (0.88 to 1.13)
Moroccan origin	1.15 (1.04 to 1.26)	1.12 (1.02 to 1.23)	0.99 (0.89 to 1.10)	1.10 (0.97 to 1.24)	1.09 (0.97 to 1.24)
Alternative medicine					
Dutch origin	Reference	Reference	Reference	Reference	Reference
South Asian Surinamese origin	0.76 (0.68 to 0.84)	0.76 (0.68 to 0.84)	0.87 (0.77 to 0.97)	0.91 (0.81 to 1.02)	0.91 (0.81 to 1.02)
African Surinamese origin	0.66 (0.59 to 0.73)	0.64 (0.57 to 0.71)	0.68 (0.61 to 0.76)	0.71 (0.63 to 0.79)	**0.71 (0.63 to 0.80)**
Ghanaian origin	0.55 (0.48 to 0.63)	0.54 (0.47 to 0.61)	0.70 (0.59 to 0.82)	0.73 (0.62 to 0.86)	**0.73 (0.62 to 0.87)**
Turkish origin	0.66 (0.59 to 0.74)	0.67 (0.59 to 0.74)	0.87 (0.77 to 0.99)	0.91 (0.79 to 1.05)	0.91 (0.79 to 1.05)
Moroccan origin	0.55 (0.49 to 0.62)	0.54 (0.48 to 0.60)	0.69 (0.60 to 0.78)	0.73 (0.63 to 0.85)	**0.73 (0.63 to 0.84)**

Bold values in Model 5 (fully adjusted model) indicate statistical significance at alpha of 0.05, defined as a 95% confidence interval excluding 1.0.

PR, prevalence ratio.

**PRs** and 95% CIs are presented for each model. The model represents a Poisson regression (log-link) with robust (sandwich) SE; pooled across imputations using Rubin’s rules.

Missing data were handled using multiple imputation (MICE); analyses were conducted on the fully imputed dataset (n=21 614).

**Bold** values indicate statistical significance at α=0.05 (95% CI excluding the null value) in fully adjusted models.

**Model 1:** unadjusted for other variables.

**Model 2:** adjusted for age and sex.

**Model 3:** adjusted for age, sex, marital status, education, occupation and health literacy.

**Model 4:** adjusted for age, sex, marital status, education, occupation, health literacy, smoking, alcohol use and physical activity.

**Model 5:** adjusted for age, sex, marital status, education, occupation, health literacy, smoking, alcohol use, physical activity, CKD, diabetes and hypertension.

The **overall sample size** is 21 614, with the following distribution by migration background: Dutch (n=4564), South-Asian Surinamese (n=3042), African Surinamese (n=4151), Ghanaian (n=2338), Turkish (n=3613) and Moroccan (n=3906).

Since GP services are not only used for illness but also for routine health checks and preventive care, we further explored how GP use varied based on self-rated health status (table 3). We found that even among those in good health, South Asian Surinamese (aPR: 1.05, 95% CI 1.01 to 1.08) and African Surinamese (aPR: 1.04, 95% CI 1.01 to 1.07) individuals continued to show higher GP utilisation compared with those of Dutch origin. Notably, African Surinamese individuals in very good or excellent health demonstrated even higher GP use (aPR: 1.10, 95% CI 1.04 to 1.16).

**Table 3:** Associations between migration background and use of general practitioner services by self-rated health status

	Model 1	Model 2	Model 3
	PR (95% CI)	PR (95% CI)	PR (95% CI)
Fair/poor health (n=5469)			
Dutch origin	Reference	Reference	Reference
South Asian Surinamese origin	1.07 (1.03 to 1.11)	1.07 (1.03 to 1.11)	**1.08 (1.04 to 1.12)**
African Surinamese origin	1.07 (1.03 to 1.11)	1.06 (1.02 to 1.10)	**1.06 (1.02 to 1.11)**
Ghanaian origin	1.02 (0.97 to 1.07)	1.02 (0.97 to 1.07)	1.03 (0.98 to 1.08)
Turkish origin	1.01 (0.97 to 1.05)	1.02 (0.97 to 1.06)	1.03 (0.99 to 1.08)
Moroccan origin	1.03 (0.99 to 1.07)	1.03 (0.99 to 1.07)	1.04 (0.99 to 1.09)
Good health (n=11 084)			
Dutch origin	Reference	Reference	Reference
South Asian Surinamese origin	1.03 (0.99 to 1.06)	1.04 (1.01 to 1.07)	**1.05 (1.02 to 1.08)**
African Surinamese origin	1.04 (1.01 to 1.07)	1.03 (1.00 to 1.06)	**1.04 (1.01 to 1.07)**
Ghanaian origin	1.00 (0.97 to 1.04)	1.00 (0.97 to 1.04)	1.01 (0.97 to 1.05)
Turkish origin	0.96 (0.93 to 1.00)	0.99 (0.96 to 1.02)	1.01 (0.98 to 1.05)
Moroccan origin	0.95 (0.92 to 0.98)	0.97 (0.94 to 1.00)	0.98 (0.95 to 1.02)
Very good/excellent health (n=5001)			
Dutch origin	Reference	Reference	Reference
South Asian Surinamese origin	1.02 (0.96 to 1.09)	1.04 (0.98 to 1.11)	1.05 (0.99 to 1.13)
African Surinamese origin	1.09 (1.04 to 1.15)	1.10 (1.04 to 1.15)	**1.10 (1.04 to 1.16)**
Ghanaian origin	1.04 (0.98 to 1.10)	1.04 (0.98 to 1.09)	1.04 (0.96 to 1.13)
Turkish origin	0.93 (0.86 to 1.00)	0.98 (0.91 to 1.05)	0.98 (0.91 to 1.06)
Moroccan origin	0.96 (0.90 to 1.03)	1.00 (0.93 to 1.07)	1.00 (0.93 to 1.07)

Bold values in Model 3 (fully adjusted model) indicate statistical significance at alpha of 0.05, defined as a 95% confidence interval excluding 1.0.

PR, prevalence ratio.

**PRs** and 95% CIs are presented for each model. The model represents a Poisson regression (log-link) with robust (sandwich) SE; pooled across imputations using Rubin’s rules.

Missing data were handled using multiple imputation (MICE); analyses were conducted on the fully imputed dataset (n=21 614).

**Bold values** in Model 3 (fully adjusted model) indicate statistical significance at α=0.05, defined as a 95% CI excluding 1.0.

**Model 1:** unadjusted for other variables.

**Model 2:** adjusted for age and sex.

**Model 3:** adjusted for age, sex, education, occupation and health literacy.

**Self-rated health status** was assessed using the first item of the SF-12, a widely used general health questionnaire. Participants were asked: ‘In general, would you say your health is: excellent, very good, good, fair or poor?’ Responses were categorised into three groups: excellent/very good, good and fair/poor.

**Fair/poor: Dutch:** n=430, South-Asian Surinamese: n=944, African Surinamese: n=935, Ghanaian: n=470, Turkish: n=1235, Moroccan: n=1472.

**Good: Dutch:** n=2211, South-Asian Surinamese: n=1624, African Surinamese: n=2341, Ghanaian: n=1184, Turkish: n=1877, Moroccan: n=1883.

**Excellent/very good:** Dutch: n=1923, South-Asian Surinamese: n=474, African Surinamese: n=875, Ghanaian: n=684, Turkish: n=501, Moroccan: n=551.

### Migration background and specialist services use

We found that individuals of South Asian Surinamese (aPR: 1.11, 95% CI 1.06 to 1.16), African Surinamese origin (aPR: 1.07, 95% CI 1.03 to 1.12) and Turkish origin (aPR: 1.14, 95% CI 1.08 to 1.20) are more likely to use these services compared with their Dutch counterparts. Conversely, we found that individuals of Ghanaian origin (aPR: 0.85, 95% CI 0.80 to 0.91) demonstrate a lower likelihood of using medical specialist services. Moroccan origin (aPR: 1.03, 95% CI 0.94 to 1.12) show usage patterns like the Dutch population (table 2, online supplemental appendix 4).

### Migration background and allied health services use

While crude models suggest increased use of allied health services among most migrant groups compared with the Dutch, this difference diminishes when factors like socioeconomic status, health behaviours, health literacy and chronic diseases are considered. In the fully adjusted model, we found that South Asian Surinamese (aPR: 1.09, 95% CI 0.98 to 1.21), African Surinamese (aPR: 1.02, 95% CI 0.88 to 1.13), Turkish (PR: 1.00, 95% CI 0.88 to 1.13) and Moroccans (aPR: 1.09, 95% CI 0.97 to 1.24) have the same use of allied health services compared with the Dutch population, while the use of services by Ghanaians is lower than the Dutch (Ghanaian aPR: 0.84, 95% CI 0.73 to 0.97; table 2, online supplemental appendix 4).

### Migration background and alternative healthcare use

We found lower use among migrant groups, even after accounting for confounding variables like socioeconomic factors, health behaviours, health literacy and comorbidities. In the fully adjusted model, we found that African Surinamese (PR: 0.71, 95% CI 0.63 to 0.80), Ghanaian origin participants (aPR: 0.73, 95% CI 0.62 to 0.87) and Moroccan (aPR: 0.73, 95% CI 0.63 to 0.87) individuals show lower use compared with the Dutch, while Turkish (aPR: 0.91, 95% CI 0.79 to 1.05) and South Asian Surinamese (aPR: 0.91, 95% CI 0.81 to 1.02) individuals have similar use patterns (table 2, online supplemental appendix 4).

### Potential reasons for seeking care

As the exact medical reasons for seeking care were not provided, we used proxy measures in exploratory analyses to gain insights. For GP visits, apart from self-rated health status (table 3), we also assessed whether presence of diabetes or hypertension (common chronic non-communicable diseases) influenced GP utilisation. We observed the same pattern of higher migrant groups’ GP service use than the Dutch individuals as in the primary analysis (online supplemental appendix 5).

For specialists, allied health services and complementary medicine, we analysed the type of service used to infer possible medical reasons. Overall, we found that certain services, such as those provided by cardiologists, ophthalmologists, dermatologists, psychologists and manual therapists, were commonly used across all study groups. However, considerable variation existed in the use of other services, as detailed in online supplemental appendix 6–9.

### Sensitivity analyses

In sensitivity analyses, primary models were repeated using the unimputed (complete-case) dataset and compared with the imputed analyses. Results were similar between complete-case and imputed analyses in both magnitude and direction, supporting the robustness of the conclusions to the missing-data approach (online supplemental appendix 10).

## Discussion

### Key findings

We examined healthcare utilisation patterns across migrant groups in Amsterdam and found that most groups demonstrated similar or higher use of GP, specialist and allied health services compared with Dutch-origin individuals. Ghanaian-origin participants differed in showing comparable GP use but lower specialist and allied health utilisation.

### Discussion of key findings

The relatively high utilisation of mainstream healthcare services (GP, specialist, allied) among most migrant groups—except for Ghanaians, who exhibited low specialist and allied healthcare use—is noteworthy. This contrasts with findings from Beveridge and Bismarck model countries,^14 15^ where migrants have been reported to have significantly lower overall healthcare access.^18^,^22^ In our study, most migrant groups appear to be effectively using this healthcare system in Amsterdam within the Netherlands dual/hybrid healthcare setting.

It is possible that both population-level determinants and system-level factors contributed to the observed patterns. However, our study examined only population-level characteristics, and system-level influences could only be inferred rather than directly tested. Regarding population-level determinants, we hypothesised that factors such as acculturation, language proficiency, health literacy and lifestyle characteristics would explain the increased utilisation observed among migrant groups.^8 9^ However, adjusting for these variables did not significantly alter the findings. Additionally, use of GP services was equal to or higher among migrant groups than the Dutch participants irrespective of self-reported health status, suggesting that poor health status among most migrants was not the only driver of higher or equal use, but also in good health. Beyond these individual and population-level characteristics, system-level mechanisms may also potentially play a role. For example, mandatory basic health insurance may potentially be a key driver, as it ensures coverage while providing government assistance for low-income individuals.^23^ Another potential factor could be that healthcare quality in the Netherlands may be perceived as superior to that in migrants’ countries of origin, thus motivating healthcare access, as access is universal even for those with low incomes.^23^ Nevertheless, our study could not directly test these structural mechanisms. Future research should explicitly investigate system-level determinants of healthcare utilisation, including insurance design, financial incentives, perceived quality of care and broader institutional characteristics.

While reasons for service use varied, consultations with cardiologists, dermatologists and ophthalmologists were commonly observed across all groups. Certain migrant groups, like the Turkish population, displayed notably higher use of neurology services, while the Moroccan population had notably higher use of radiology services. This could reflect specific health needs within these groups, highlighting that migrant populations are generally able to access any type of healthcare service when needed. Although our study did not include direct measures of system-level determinants, several structural factors may potentially be at play. For example, because GPs function as gatekeepers in the Dutch healthcare system, referral practices may potentially facilitate specialist access across migrant groups.^23^ Other structural characteristics—such as geographical proximity to services, waiting times, ease of obtaining appointments and insurance-related financial arrangements—may also potentially influence specialist use.^23^ Additionally, differences in specialist use may also potentially reflect culturally shaped health beliefs, disease prioritisation within communities, awareness of available services through social networks or differential referral practices. However, as these system-level variables were not measured in the HELIUS cohort, their role remains uncertain and should be carefully examined in future research.

In contrast, complementary medicine usage was lower among migrant groups, aligning with findings in other European countries.^14 15 29^ Services like acupuncture and homeopathy are typically not covered by the basic insurance package, necessitating out-of-pocket payments, which could potentially discourage use.^23^ However, this pattern persisted even after controlling for socioeconomic status. Other unmeasured factors may also be at play here. For example, unfamiliarity has the potential to play a role, as such services might not be prevalent or culturally ingrained in migrants’ countries of origin.^30^ Although traditional and natural healing practices are integral to the cultures of some migrant groups, such as Ghanaians, and might be expected to supplement healthcare use, these practices were not widely used among other migrant populations in the study.^30^ This could potentially indicate variations in cultural ties to complementary medicine or adoption of mainstream services as the main form of healthcare, like how migrants adopt Western diets and lifestyles when they migrate.^30^ In-depth qualitative studies are needed to understand these allied healthcare use patterns.

Surprisingly, Ghanaians showed lower utilisation of medical specialist and allied services than the other migrant groups, a trend that persisted even after accounting for factors such as demographics, lifestyle, health literacy, acculturation and chronic conditions. These findings merit further investigation, as they may reflect unique barriers faced by this group in accessing past primary care (GPs). In-depth qualitative studies could provide answers.

The findings from this study carry important implications for public health and clinical practice, particularly in the development of healthcare models that promote equitable access for migrant populations. Within Amsterdam, most migrant groups demonstrated comparable or higher utilisation of mainstream healthcare services relative to Dutch-origin individuals. Further research within this context may help identify structural and organisational features that facilitate such access, thereby providing transferable lessons for improving mainstream healthcare use among migrant populations in other settings.

### Strengths and limitations

This study benefits from a large, diverse sample from the HELIUS dataset, allowing for generalisable insights into healthcare utilisation across multiple migrant groups in the Netherlands. The study has comprehensive data on socioeconomic status, health literacy, language proficiency, acculturation levels and lifestyle factors which are seen as barriers to care helping to explain effects of migration background on healthcare utilisation. However, reliance on self-reported data introduces the possibility of recall bias, particularly for infrequent services like complementary medicine. Furthermore, the qualitative aspects of healthcare access, such as the specific reasons for service use and the enablers and barriers experienced by different groups, were not captured in this study. Future research incorporating qualitative data could provide a more nuanced understanding of the motivations and challenges influencing migrant healthcare behaviours. Additionally, migration background was defined using country-of-birth criteria for participants and their parents. As such, individuals born in the Netherlands with both parents also born in the Netherlands may have non-European ancestry. However, such individuals would largely represent third-generation migrants. Given that large-scale Surinamese migration to the Netherlands began in the mid-1970s, third-generation adults within the studied age range were likely few during the baseline recruitment period (2011–2015, pre-COVID-19); therefore, any resulting misclassification is expected to be minimal. Because the HELIUS cohort was recruited exclusively in Amsterdam, findings may not fully represent healthcare utilisation patterns in other Dutch regions.^31^

### Conclusion

Healthcare utilisation across GP, specialist and allied health services within Amsterdam, operating under the Netherlands’ dual/hybrid healthcare model, was comparable to or higher among most migrant groups relative to Dutch-origin individuals and contrasts with reports of lower utilisation in some other European healthcare settings. Further research is needed to better understand the structural and contextual factors underlying these patterns and to inform efforts aimed at improving access for diverse populations in other European contexts.

## Supplementary material

10.1136/bmjgh-2025-020024 online supplemental file 1

## Data Availability

Data are available upon reasonable request.
