# Supplementary material for: Healthcare utilisation among migrants in the Netherlands’ unique hybrid healthcare system: the HELIUS study
Source: BMJ Glob Health. 2026 Mar 16;11(3):e020024. doi: 10.1136/bmjgh-2025-020024 (PMC12993351; doi:10.1136/bmjgh-2025-020024)
Supplement: online supplemental file 1 [file bmjgh-11-3-s001.pdf]

SECTION F – HEALTH INFORMATION AND USE OF CARE

32 Folders and forms

We're interested to know whether, in general, folders and letters containing medical information can be understood.

32.1 How often do you have someone help you read hospital materials?

- ☐ Never
- ☐ Occasionally
- ☐ Sometimes
- ☐ Often
- ☐ Always
- ☐ Doesn't apply

32.2 How confident are you about filling in medical forms by yourself?

- ☐ Extremely confident
- ☐ Quite confident
- ☐ Somewhat confident
- ☐ Not very confident
- ☐ Not at all confident
- ☐ Doesn't apply

32.3 How often do you have problems learning more about your medical condition because it's hard for you to understand written information?

- ☐ Never
- ☐ Occasionally
- ☐ Sometimes
- ☐ Often
- ☐ Always

33 Use of health care

33.1 When was the last time you consulted a general practitioner (GP/family doctor/'huisarts') for yourself?

This includes all visits to the doctor's practice or visits by the doctor to you at home, as well as consultations over the telephone. Include contact(s) with someone who was replacing your own doctor. Do not include telephone contact(s) with the assistant to make an appointment or get a prescription refilled.

- ☐ Never → **Go to Question 33.4**
- ☐ 12 months ago or more → **Go to Question 33.3**
- ☐ More than 2 months but less than 12 months ago → **Go to Question 33.3**
- ☐ In the past 2 months

33.2 How many times in the past 2 months have you consulted your general practitioner? |__|__| times

33.3 How satisfied are you with your general practitioner?

- ☐ Dissatisfied
- ☐ Slightly dissatisfied
- ☐ Reasonably satisfied
- ☐ Quite satisfied
- ☐ Very satisfied

33.4 Which of the following medical specialists have you visited for yourself in the past 12 months?
You can give more than one answer/specialist.

Do not include specialist help during a hospital stay or telephone contact with the secretary or assistant.
Do include treatment at outpatient clinics, scheduled check-ups, telephone consultations, emergency department visits, and X-rays.

- ☐ Allergoloog (allergy specialist)
- ☐ Cardioloog (cardiologist)
- ☐ Chirurg (surgeon)
- ☐ Dermatoloog (dermatologist)
- ☐ Gastro-enteroloog/maag-darm-lever arts (gastro-enterologist)
- ☐ Gynaecoloog/vrouwenarts (gynaecologist)
- ☐ Internist (internal medicine specialist)
- ☐ KNO-arts/ keel-neus-oor arts (ear, nose, and throat specialist)
- ☐ Longarts (lung specialist)
- ☐ Mond- en kaakchirurg (oral surgeon)
- ☐ Neuroloog (neurologist)
- ☐ Oogarts (eye specialist)
- ☐ Orthopeed (orthopaedic specialist)
- ☐ Plastisch chirurg (plastic surgeon)
- ☐ Psychiater (psychiatrist)
- ☐ Radioloog (radiologist)
- ☐ Reumatoloog (rheumatologist)
- ☐ Revalidatiearts (rehabilitation doctor)
- ☐ Uroloog (urologist)
- ☐ Other medical specialist, namely:

  ………………………………………………………………
- ☐ In the past 12 months I have not seen a medical specialist

33.5 Which of the following health care workers have you consulted for yourself in the past 12 months?
You can give more than one answer/health care worker.

- ☐ Social worker
- ☐ Psychologist
- ☐ Psychiatrist
- ☐ Psychotherapist
- ☐ Other psychosocial health care worker, namely:

  ………………………………………………………………
- ☐ In the past 12 months, I have not consulted a psychosocial health care worker

33.6 Which of the following alternative healers have you consulted for yourself in the past 12 months?
You can give more than one answer/alternative healer.

- ☐ Acupuncturist (not your own GP/'huisarts')
- ☐ Anthroposophical healer (not your own GP/'huisarts')
- ☐ Homeopath (not your own GP/'huisarts')
- ☐ Manual therapist (chiropractor, osteopath, manual therapist, 'bottenkraker')
- ☐ Natural healer (dietary or herbal therapy, etc.)
- ☐ Paranormal healer (clairvoyant, spiritual healer, magnetizer, etc.)
- ☐ Spiritual/religious guide (for example, an imam or priest)
- ☐ Traditional healer, namely:

  …………………………………………….(type of healer)
- ☐ Other alternative healer, namely:

  …………………………………………….(type of healer)
- ☐ In the past 12 months, I have not consulted an alternative healer

Appendix 2. Full description of Measurements obtained from HELIUS study

Demographic data collected included sex (male or female), age in years, occupational level (categorized from elementary to scientific, according to the International Standard Classification of Occupations (ISCO-08)),[1] marital status (e.g., married, cohabiting, divorced). Educational level was based on the highest qualification attained, either in Netherlands or in the country of origin, and it was categorized into four groups: (1) never been to school or elementary schooling only, (2) lower vocational schooling or lower secondary schooling, (3) intermediate vocational schooling or intermediate/secondary schooling, or (4) higher vocational schooling or university.

Lifestyle factors assessed included smoking status (current smoker, former smoker, or never smoked), alcohol consumption (yes or no), and physical activity level, measured using the SQUASH questionnaire.[2] Participants were categorized as adherent (yes/no) to the Dutch guideline for physical activity when the sum of the number of days per week for each moderate- and high-intensity activity lasting at least 30 min was greater than or equal to five.

Health literacy was assessed using the Set of Brief Screening Questions (SBSQ).[3] The three-item SBSQ evaluates reading assistance, confidence in completing medical forms, and difficulty understanding medical information. Responses were scored on a Likert scale and summed.[3] A score of ≤2 indicates inadequate health literacy, while ≥3 suggests adequate health literacy.[3] Acculturation indicators included self-reported Dutch language proficiency, and Berry's acculturation strategy (integration, assimilation, separation, or marginalization), based on attitudes and behaviors toward heritage culture and Dutch culture.[4]

We also obtained data on diabetes, hypertension, and chronic kidney disease as the common chronic non-communicable conditions available in HELIUS. Diabetes was defined using WHO criteria (fasting plasma glucose ≥7.0 mmol/L, use of anti-diabetic medications or self-reported previous doctor diagnosis). Hypertension was defined by WHO criteria (systolic blood pressure ≥140 mmHg or diastolic ≥90 mmHg) or use of anti-hypertensive medications or self-reported previous doctor diagnosis. Kidney function was evaluated using the 2021 CKD-EPI creatinine equation (race-free), with estimated glomerular filtration rate (eGFR) values categorized as normal (eGFR ≥60 mL/min) or decreased (eGFR <60 mL/min).[5]

REFERENCES

1. Ganzeboom H. International standard classification of occupations ISCO-08 with ISEI-08 scores. *Version of July* 2010; **27**: 2010.
2. Nicolaou M, Gademan M, Snijder M, et al. Validation of the SQUASH physical activity questionnaire in a multi-ethnic population: the HELIUS study. *PloS one* 2016; **11**(8): e0161066.
3. Fransen M, Van Schaik T, Twickler T, Essink-Bot M. Applicability of internationally available health literacy measures in the Netherlands. *Journal of health communication* 2011; **16**(sup3): 134-49.
4. Schwartz SJ, Zamboanga BL. Testing Berry's model of acculturation: a confirmatory latent class approach. *Cultural diversity & ethnic minority psychology* 2008; **14**(4): 275.
5. Levin A, Stevens PE, Bilous RW, et al. Kidney Disease: Improving Global Outcomes (KDIGO) CKD Work Group. KDIGO 2012 clinical practice guideline for the evaluation and management of chronic kidney disease. *Kidney international supplements* 2013; **3**(1): 1-150.

Appendix 3. Missingness map of variables before multiple imputations.

Categories	Measure	Number of missing values	Denominator	Percentage missing (%)	Imputed (Yes/No)
**Demographic measures**	Migration background	0	Out of N=21614	0.0	No
	Age (years)	0	Out of N=21614	0.0	No
	Sex	0	Out of N=21614	0.0	No
**Socioeconomic measures**	Marital status	124	Out of N=21614	0.6	Yes
	Occupational level	3,391	Out of N=21614	15.7	Yes
	Educational level	195	Out of N=21614	0.9	Yes
**Lifestyle measures**	Smoking status	99	Out of N=21614	0.5	Yes
	Alcohol consumption	119	Out of N=21614	0.6	Yes
	Physical activity levels	29	Out of N=21614	0.1	Yes
	Health literacy	100	Out of N=21614	0.5	Yes
**Clinical measures**	Kidney function (eGFR < 60)	122	Out of N=21614	0.6	Yes
	Diabetes	113	Out of N=21614	0.5	Yes
	Hypertension	51	Out of N=21614	0.2	Yes
**Acculturation factors**	Difficulty with Dutch language	120	Out of N=17050 (non-Dutch)	0.7	Yes
	Berry's Acculturation	318	Out of N=17050 (non-Dutch)	1.9	Yes
**Health status**	Self-rated health status	60	Out of N=21614	0.3	Yes
**Health service use**	General practitioner use	352	Out of N=21614	1.6	Yes

	Medical specialist use	620	Out of N=21614	2.9	Yes
	Allied health services use	762	Out of N=21614	3.5	Yes
	Alternative medicine use	917	Out of N=21614	4.2	Yes

Note: All missing values were imputed with MICE. Acculturation factors (Dutch language difficulty, and cultural orientation) are not applicable to Dutch participants. missingness for these variables is calculated among non-Dutch participants only.

Appendix 4. Use of healthcare in populations with migration background adjusted for acculturation factors

	**PR (95% CI)**
**GP use**	
South Asian Surinamese origin	**Reference**
African Surinamese origin	0.98 (0.96-1.00)
Ghanaian origin	0.94 (0.91-0.97)
Turkish origin	0.97 (0.95-1.00)
Moroccan origin	0.98 (0.96-1.00)
**Specialist use**	
South Asian Surinamese origin	Reference
African Surinamese origin	0.97 (0.93-1.01)
Ghanaian origin	**0.76 (0.71-0.81)**
Turkish origin	1.02 (0.97-1.07)
Moroccan origin	**0.93 (0.89-0.98)**
**Allied health use**	
South Asian Surinamese origin	Reference
African Surinamese origin	0.95 (0.85-1.05)
Ghanaian origin	**0.71 (0.62-0.81)**
Turkish origin	**0.87 (0.78-0.98)**
Moroccan origin	1.00 (0.89-1.12)
**Alternative health use**	
South Asian Surinamese origin	Reference
African Surinamese origin	**0.79 (0.70-0.90)**
Ghanaian origin	**0.78 (0.65-0.92)**
Turkish origin	0.99 (0.86-1.15)
Moroccan origin	**0.82 (0.70-0.95)**

Prevalence ratios and 95% confidence intervals are presented for each model. The model represents a Poisson regression (log-link) with robust (sandwich) SE; pooled across imputations using Rubin's rules; of the relationship between migration background and healthcare use, adjusted for age, sex, marital status, education, occupation, health literacy, smoking, alcohol use, physical activity, chronic kidney disease, diabetes, hypertension, and acculturation factors.

Missing data were handled using multiple imputation (MICE); analyses were conducted on the fully imputed dataset (N= 21614)

Bold values in fully adjusted model indicate statistical significance at α = 0.05, defined as a 95% confidence interval excluding 1.0.

The **overall sample** size is 21,614, with the following distribution by migration background : Dutch (N=4,564), South-Asian Surinamese (N=3,042), African Surinamese (N=4,151), Ghanaian (N=2,338), Turkish (N=3,613), and Moroccan (N=3,906).

Appendix 5. Associations between migration background and use of GP services in those with cardiometabolic diseases

	Model 1	Model 2	Model 3	Model 4
	PR (95%CI)	PR (95%CI)	PR (95%CI)	PR (95%CI)
**Participants with both hypertension and diabetes (N=8660)**				
Dutch origin	Reference	Reference	Reference	Reference
South Asian Surinamese origin	1.14 (1.11-1.18)	1.14 (1.10-1.17)	1.12 (1.08-1.16)	**1.13 (1.09-1.17)**
African Surinamese origin	1.12 (1.08-1.15)	1.10 (1.07-1.13)	1.09 (1.06-1.13)	**1.10 (1.06-1.13)**
Ghanaian origin	1.06 (1.03-1.10)	1.07 (1.03-1.11)	1.04 (1.00-1.09)	**1.05 (1.01-1.10)**
Turkish origin	1.06 (1.02-1.10)	1.08 (1.04-1.12)	1.05 (1.01-1.10)	**1.05 (1.01-1.10)**
Moroccan origin	1.09 (1.05-1.13)	1.10 (1.06-1.14)	1.08 (1.03-1.12)	**1.08 (1.04-1.13)**
**Hypertension only (N=8069)**				
Dutch origin	Reference	Reference	Reference	Reference
South Asian Surinamese origin	1.14 (1.11-1.18)	1.14 (1.10-1.17)	1.12 (1.08-1.16)	**1.13 (1.09-1.17)**
African Surinamese origin	1.12 (1.08-1.15)	1.10 (1.07-1.14)	1.09 (1.06-1.13)	**1.10 (1.06-1.14)**
Ghanaian origin	1.07 (1.03-1.11)	1.07 (1.04-1.11)	1.04 (1.00-1.09)	**1.05 (1.01-1.10)**
Turkish origin	1.05 (1.01-1.09)	1.07 (1.03-1.12)	1.04 (1.00-1.09)	**1.05 (1.00-1.10)**
Moroccan origin	1.08 (1.04-1.13)	1.09 (1.05-1.14)	1.07 (1.02-1.12)	**1.08 (1.03-1.13)**
**Diabetes only (N=2343)**				
Dutch origin	Reference	Reference	Reference	Reference
South Asian Surinamese origin	1.12 (1.05-1.21)	1.13 (1.05-1.22)	1.13 (1.05-1.22)	**1.14 (1.05-1.23)**
African Surinamese origin	1.10 (1.02-1.19)	1.10 (1.02-1.18)	1.09 (1.01-1.18)	**1.10 (1.02-1.18)**
Ghanaian origin	1.08 (1.00-1.17)	1.10 (1.02-1.20)	1.10 (1.00-1.20)	**1.10 (1.01-1.20)**
Turkish origin	1.09 (1.01-1.17)	1.11 (1.03-1.20)	1.11 (1.02-1.21)	**1.11 (1.02-1.21)**
Moroccan origin	1.10 (1.02-1.18)	1.10 (1.02-1.19)	1.11 (1.02-1.20)	**1.10 (1.01-1.20)**

Prevalence ratios (PRs) and 95% confidence intervals are presented for each model. The model represents a Poisson regression (log-link) with robust (sandwich) SE; pooled across imputations using Rubin's rules.

Missing data were handled using multiple imputation (MICE); analyses were conducted on the fully imputed dataset (N= 21614)

Bold values in fully adjusted model indicate statistical significance at α = 0.05, defined as a 95% confidence interval excluding 1.0.

Model 1, unadjusted for other variables.

Model 2, adjusted for age and sex.

Model 3, adjusted for age, sex, marital status, education, occupation, and health literacy.

Model 4 adjusted for age, sex, marital status, education, occupation, health literacy, smoking, alcohol use, and physical activity.

Hypertension or diabetes: The combined counts of participants with hypertension or diabetes were as follows: Dutch origin: 1,301; South Asian Surinamese: 1,742; African Surinamese: 2,397; Ghanaian: 1,499; Turkish: 1,270; and Moroccan origin: 1,161 (total N = 8,660).

Hypertension: A total of 8,069 participants had hypertension. By migration background, the counts were as follows: Dutch origin: 1,354; South Asian Surinamese: 1,293; African Surinamese: 2,098; Ghanaian: 1,311; Turkish: 1,060; and Moroccan origin: 953.

Diabetes: Among participants, 2,343 individuals were diagnosed with diabetes. By migration background, the counts were as follows: Dutch origin: 165; South Asian Surinamese: 592; African Surinamese: 498; Ghanaian: 273; Turkish: 370; and Moroccan origin: 445.

Appendix 6. Underlying reasons for use of care

Specialist services

The primary reasons for specialist consultations were consistent across groups, with eye specialists (9%), dermatologists (7%), and cardiologists (7%) being the most sought-after. However, some variations were observed. The Dutch were more inclined to visit gynecologists (6%) and orthopedic specialists (7%). South Asian Surinamese individuals showed a higher likelihood of consulting internal medicine specialists (7%) and ear, nose, and throat specialists (7%). African Surinamese individuals were more likely to consult gynecologists (8%) and internal medicine specialists (8%). Ghanaians primarily sought care from gynecologists (8%). Turkish individuals showed a higher tendency to visit neurologists (8%), orthopedic specialists (7%), and psychiatrists (8%). Lastly, Moroccans were more likely to consult radiologists (7%) and neurologists (6%) (Appendix 7).

Allied health services

The most common reason for allied health visits was to see a psychologist (8%). However, Ghanaians were more likely to see social workers (7%), with only 2% consulting psychologists. African Surinamese individuals also showed a higher tendency to see social workers (8%) in addition to psychologists (7%) (Appendix 8).

Alternative health services.

The most common reason for alternative health service visits was for manual therapy (5.1%). However, South Asian Surinamese (3.3%) and Turkish (3.6%) individuals additionally used acupuncture services more, while Ghanaians used religious/spiritual services. (Appendix 9).

Appendix 7: Reason for use of specialist care

Reason for specialist visit	Total<br>N=21,614	Dutch Origin<br>N=4564	South-Asian Surinamese Origin<br>N=3042	African Surinamese Origin<br>N =4151	Ghanaian Origin<br>N= 2338	Turkish Origin<br>N =3613	Moroccan Origin<br>N =3906	P-value
	n (%)	n (%)	n (%)	n (%)	n (%)	n (%)	n (%)	
Allergy specialist	216 (1.0)	27 (0.6)	28 (0.9)	33 (0.8)	29 (1.2)	63 (1.7)	36 (0.9)	P<0.001
Cardiologist	1,434 (6.6)	229 (5.0)	338 (11.1)	315 (7.6)	114 (4.9)	261 (7.2)	177 (4.5)	P<0.001
Surgeon	856 (4.0)	189 (4.1)	138 (4.5)	221 (5.3)	57 (2.4)	103 (2.9)	148 (3.8)	P<0.001
Dermatologist	1,573 (7.3)	342 (7.5)	331 (10.9)	285 (6.9)	86 (3.7)	264 (7.3)	265 (6.8)	P<0.001
Gastroenterologist	931 (4.3)	160 (3.5)	123 (4.0)	185 (4.5)	57 (2.4)	236 (6.5)	170 (4.4)	P<0.001
Gynecologist	1,388 (6.4)	277 (6.1)	178 (5.9)	310 (7.5)	176 (7.5)	233 (6.4)	214 (5.5)	P<0.001
Internal medicine	1,143 (5.3)	197 (4.3)	223 (7.3)	319 (7.7)	93 (4.0)	160 (4.4)	151 (3.9)	P<0.001
Ear, nose, and throat	1,231 (5.7)	220 (4.8)	207 (6.8)	223 (5.4)	76 (3.3)	298 (8.2)	207 (5.3)	P<0.001
Lung specialist	672 (3.1)	103 (2.3)	147 (4.8)	145 (3.5)	31 (1.3)	147 (4.1)	99 (2.5)	P<0.001
Oral surgeon	765 (3.5)	123 (2.7)	140 (4.6)	253 (6.1)	44 (1.9)	94 (2.6)	111 (2.8)	P<0.001
Neurologist	1,200 (5.6)	170 (3.7)	187 (6.1)	261 (6.3)	85 (3.6)	272 (7.5)	225 (5.8)	P<0.001
Eye specialist	1,985 (9.2)	321 (7.0)	433 (14.2)	536 (12.9)	153 (6.5)	261 (7.2)	281 (7.2)	P<0.001
Orthopedic specialist	1,339 (6.2)	303 (6.6)	176 (5.8)	300 (7.2)	83 (3.6)	267 (7.4)	210 (5.4)	P<0.001
Plastic surgeon	262 (1.2)	59 (1.3)	32 (1.1)	76 (1.8)	10 (0.4)	50 (1.4)	35 (0.9)	P<0.001
Psychiatrist	937 (4.3)	140 (3.1)	150 (4.9)	136 (3.3)	30 (1.3)	272 (7.5)	209 (5.4)	P<0.001
Radiologist	1,255 (5.8)	266 (5.8)	181 (6.0)	269 (6.5)	82 (3.5)	185 (5.1)	272 (7.0)	P<0.001
Rheumatologist	486 (2.2)	54 (1.2)	107 (3.5)	111 (2.7)	23 (1.0)	99 (2.7)	92 (2.4)	P<0.001
Rehabilitation doctor	244 (1.1)	37 (0.8)	47 (1.5)	54 (1.3)	20 (0.9)	37 (1.0)	49 (1.3)	P<0.001
Urologist	563 (2.6)	125 (2.7)	75 (2.5)	115 (2.8)	28 (1.2)	124 (3.4)	96 (2.5)	P<0.001
Other medical	1,484 (6.9)	336 (7.4)	204 (6.7)	278 (6.7)	141 (6.0)	264 (7.3)	261 (6.7)	P=0.003

P-values were obtained using chi-square tests to assess the association between migration background and the likelihood of visiting each type of specialist.

Other medical specialists beyond those listed were not specifically named in the questionnaire and are grouped under "Other medical specialists."

Missing data were handled using multiple imputation (MICE); analyses were conducted on the fully imputed dataset (N= 21614)

Appendix 8. Reason for use of allied health services

**Reason for Visit**	**Total**<br><br>**(N=21,614)**	**Dutch Origin**<br><br>**(N=4,564)**	**South-Asian Surinamese Origin**<br><br>**(N=3,042)**	**African Surinamese Origin**<br><br>**(N=4,151)**	**Ghanaian Origin**<br><br>**(N=2,338)**	**Turkish Origin**<br><br>**(N=3,613)**	**Moroccan Origin**<br><br>**(N=3,906)**	**P-value**
	**n (%)**	**n (%)**	**n (%)**	**n (%)**	**n (%)**	**n (%)**	**n (%)**	
Social worker	1,080 (5.0%)	77 (1.7%)	170 (5.6%)	322 (7.8%)	164 (7.0%)	138 (3.8%)	209 (5.4%)	P < 0.001
Psychologist	1,741 (8.1%)	369 (8.1%)	265 (8.7%)	308 (7.4%)	45 (1.9%)	408 (11.3%)	346 (8.9%)	P < 0.001
Psychiatrist	873 (4.0%)	135 (3.0%)	148 (4.9%)	135 (3.3%)	36 (1.5%)	226 (6.3%)	193 (4.9%)	P < 0.001
Physiotherapist	573 (2.7%)	155 (3.4%)	97 (3.2%)	110 (2.6%)	60 (2.6%)	66 (1.8%)	85 (2.2%)	P = 0.025
Other Allied	426 (2.0%)	123 (2.7%)	56 (1.8%)	70 (1.7%)	68 (2.9%)	39 (1.1%)	70 (1.8%)	P = 0.036

P-values were obtained using chi-square tests to assess the association between migration background and the likelihood of visiting each type of specialist.

Other allied health care use includes visits to health professionals not specifically listed in the questionnaire.

Missing data were handled using multiple imputation (MICE); analyses were conducted on the fully imputed dataset (N= 21614)

Appendix 9. Reason for use of alternative health care

Reason for Visit	Total<br>N=21,614	Dutch Origin<br>N=4,564	South-Asian Surinamese Origin<br>N=3,042	African Surinamese Origin<br>N=4,151	Ghanaian Origin<br>N=2,338	Turkish Origin<br>N=3,613	Moroccan Origin<br>N=3,906	P-value
	n (%)	n (%)	n (%)	n (%)	n (%)	n (%)	n (%)	
Acupuncturist	634 (2.9)	183 (4.0)	101 (3.3)	98 (2.4)	31 (1.3)	131 (3.6)	90 (2.3)	P < 0.001
Anthropological	22 (0.1)	7 (0.2)	3 (0.1)	2 (0.0)	4 (0.2)	3 (0.1)	3 (0.1)	P < 0.001
Homeopath	126 (0.6)	55 (1.2)	16 (0.5)	28 (0.7)	13 (0.6)	7 (0.2)	7 (0.2)	P < 0.001
Manual therapist	1,099 (5.1)	416 (9.1)	167 (5.5)	176 (4.2)	38 (1.6)	168 (4.6)	134 (3.4)	P < 0.001
Natural healer	214 (1.0)	72 (1.6)	21 (0.7)	36 (0.9)	29 (1.2)	29 (0.8)	27 (0.7)	P < 0.001
Paranormal	156 (0.7)	51 (1.1)	31 (1.0)	57 (1.4)	6 (0.3)	3 (0.1)	8 (0.2)	P < 0.001
Spiritual/religious	334 (1.5)	15 (0.3)	76 (2.5)	64 (1.5)	77 (3.3)	34 (0.9)	68 (1.7)	P < 0.001
Traditional healer	148 (0.7)	15 (0.3)	13 (0.4)	40 (1.0)	30 (1.3)	22 (0.6)	28 (0.7)	P < 0.001
Other alternative	549 (2.5)	198 (4.3)	58 (1.9)	83 (2.0)	22 (0.9)	101 (2.8)	87 (2.2)	P < 0.001

P-values were obtained using chi-square tests to assess the association between migration background and the likelihood of visiting each type of specialist.

Other alternative health care use includes visits to health professionals not specifically listed in the questionnaire.

Missing data were handled using multiple imputation (MICE); analyses were conducted on the fully imputed dataset (N= 21614).

Appendix 10. Associations between migration background and use of health services (complete case analysis)

	Model 1	Model 2	Model 3	Model 4	Model 5
	**PR (95%CI)**	**PR (95%CI)**	**PR (95%CI)**	**PR (95%CI)**	**PR (95%CI)**
**General practitioner use**					
**N (complete-case)**	**21262**	**21262**	**17829**	**17733**	**17599**
Dutch origin	Reference	Reference	Reference	Reference	Reference
South Asian Surinamese origin	1.10 (1.08-1.13)	1.10 (1.08-1.13)	1.09 (1.06-1.12)	1.10 (1.08-1.13)	**1.09 (1.06-1.12)**
African Surinamese origin	1.10 (1.08-1.13)	1.08 (1.06-1.11)	1.07 (1.05-1.10)	1.08 (1.06-1.11)	**1.07 (1.04-1.09)**
Ghanaian origin	1.04 (1.02-1.07)	1.04 (1.01-1.06)	1.01 (0.98-1.05)	1.03 (0.99-1.06)	1.01 (0.98-1.05)
Turkish origin	1.04 (1.01-1.06)	1.06 (1.04-1.09)	1.05 (1.02-1.08)	1.05 (1.02-1.09)	**1.05 (1.02-1.08)**
Moroccan origin	1.05 (1.02-1.07)	1.06 (1.04-1.08)	1.05 (1.02-1.08)	1.06 (1.03-1.10)	**1.06 (1.03-1.10)**
**Medical specialist use**					
**N (complete-case)**	**20994**	**20994**	**17619**	**17524**	**17394**
Dutch origin	Reference	Reference	Reference	Reference	Reference
South Asian Surinamese origin	1.16 (1.11-1.21)	1.17 (1.12-1.21)	1.13 (1.08-1.18)	1.15 (1.10-1.21)	**1.11 (1.06-1.17)**
African Surinamese origin	1.15 (1.11-1.20)	1.12 (1.08-1.17)	1.10 (1.06-1.15)	1.12 (1.07-1.17)	**1.09 (1.04-1.14)**
Ghanaian origin	0.86 (0.82-0.91)	0.87 (0.82-0.92)	0.84 (0.78-0.90)	0.87 (0.81-0.93)	**0.84 (0.78-0.90)**
Turkish origin	1.11 (1.06-1.15)	1.17 (1.13-1.22)	1.16 (1.10-1.23)	1.15 (1.09-1.22)	**1.14 (1.08-1.21)**
Moroccan origin	1.00 (0.96-1.04)	1.05 (1.01-1.09)	1.06 (1.00-1.12)	1.06 (1.00-1.13)	1.06 (0.99-1.12)
**Allied health services use**					
**N (complete-case)**	**20852**	**20852**	**17537**	**17444**	**17311**
Dutch origin	Reference	Reference	Reference	Reference	Reference
South Asian Surinamese origin	1.21 (1.10-1.34)	1.21 (1.10-1.34)	1.01 (0.90-1.13)	1.09 (0.97-1.22)	1.07 (0.95-1.20)
African Surinamese origin	1.17 (1.06-1.28)	1.14 (1.04-1.26)	0.97 (0.87-1.07)	1.01 (0.91-1.13)	0.99 (0.89-1.10)
Ghanaian origin	1.01 (0.90-1.14)	0.99 (0.88-1.11)	0.61 (0.52-0.72)	0.74 (0.62-0.87)	**0.72 (0.61-0.85)**
Turkish origin	1.16 (1.06-1.28)	1.15 (1.05-1.27)	1.03 (0.91-1.17)	1.04 (0.91-1.19)	1.05 (0.92-1.20)
Moroccan origin	1.15 (1.04-1.27)	1.12 (1.02-1.23)	1.03 (0.91-1.15)	1.14 (1.00-1.31)	1.15 (0.99-1.31)
**Alternative medicine**					
**N (complete-case)**	**20697**	**20697**	**17433**	**17344**	**17213**
Dutch origin	Reference	Reference	Reference	Reference	Reference
South Asian Surinamese origin	0.75 (0.68-0.84)	0.75 (0.67-0.84)	0.85 (0.76-0.96)	0.90 (0.80-1.02)	0.91 (0.80-1.03)
African Surinamese origin	0.65 (0.59-0.73)	0.63 (0.57-0.70)	0.66 (0.59-0.74)	0.69 (0.61-0.77)	**0.69 (0.61-0.78)**
Ghanaian origin	0.54 (0.47-0.63)	0.53 (0.46-0.61)	0.64 (0.54-0.77)	0.68 (0.57-0.82)	**0.69 (0.57-0.83)**
Turkish origin	0.66 (0.59-0.74)	0.67 (0.60-0.74)	0.85 (0.74-0.97)	0.90 (0.77-1.04)	0.90 (0.78-1.05)
Moroccan origin	0.55 (0.49-0.61)	0.54 (0.48-0.60)	0.68 (0.59-0.78)	0.74 (0.63-0.87)	**0.73 (0.63-0.86)**

Prevalence ratios (PRs) and 95% confidence intervals are presented for each model. The model represents a Poisson regression (log-link) with robust (sandwich) SE;

The analyses are for complete case analyses .

Bold values indicate statistical significance at α = 0.05 (95% confidence interval excluding the null value) in fully adjusted models.

Model 1, unadjusted for other variables.

Model 2, adjusted for age and sex.

Model 3, adjusted for age, sex, marital status, education, occupation, and health literacy.

Model 4 adjusted for age, sex, marital status, education, occupation, health literacy, smoking, alcohol use, physical activity.

Model 5, adjusted for age, sex, marital status, education, occupation, health literacy, smoking, alcohol use, physical activity, chronic kidney disease, diabetes, and hypertension.
